# Ruptured carotid artery pseudoaneurysm following excision of a carotid body tumor: a rare complication managed successfully with surgery and literature review

**DOI:** 10.1590/1677-5449.202301702

**Published:** 2024-08-09

**Authors:** Basil Babu, Divij Jayant, Arunanshu Behera, Cherring Tandup, Swapnesh Kumar Sahoo, Vipul Thakur

**Affiliations:** 1 Post Graduate Institute of Medical Education and Research – PGIMER, Department of General Surgery, Chandigarh, India.

**Keywords:** carotid body tumor, pseudoaneurysms, carotid artery pseudoaneurysm, rare complication, tumor do corpo carotídeo, pseudoaneurismas, pseudoaneurisma da artéria carótida, complicação rara

## Abstract

A 47-year-old male presented with a right-sided Shamblin type 2 carotid body tumor measuring 5*5 cm. After preoperative embolization, a sub adventitial resection of the tumor was done. He was discharged after postoperative day 5 and presented again to emergency 10 days later with a bleeding pseudoaneurysm at the surgical site causing dysphagia and dyspnea. He was taken for emergency exploration of the surgical wound and, intraoperatively, it was observed that the proximal ends of the internal carotid artery and external carotid artery close to the bifurcation were forming a pseudoaneurysm, 1 cm distal to the common carotid artery. The external carotid artery was ligated and a common carotid to internal carotid artery bypass was done with a reversed saphenous vein graft. He recovered well in the postoperative period and was discharged on day 7. Pseudoaneurysm formation following carotid body tumor resection is extremely rare and has only been reported thrice in the literature.

## Resumo

Um homem de 47 anos apresentou tumor carotídeo Shamblin tipo 2 no lado direito, medindo 5 x 5 cm. Após embolização pré-operatória, foi realizada ressecção subadventicial do tumor. O paciente teve alta no quinto dia pós-operatório e voltou à emergência 10 dias depois, com pseudoaneurisma hemorrágico no sítio operatório causando disfagia e dispneia. Foi levado para exploração emergencial da ferida cirúrgica, e, no intraoperatório, 1 cm distalmente à artéria carótida comum, as extremidades proximais da artéria carótida interna e da artéria carótida externa próximas à bifurcação formavam um pseudoaneurisma. A artéria carótida externa foi ligada, e foi realizada uma ponte de safena de carótida comum para a artéria carótida interna com a veia safena invertida. O paciente se recuperou bem no pós-operatório e recebeu alta no sétimo dia. A formação de pseudoaneurisma após ressecção de tumor do corpo carotídeo é extremamente rara, tendo sido relatada apenas três vezes na literatura.

## INTRODUCTION

Carotid body tumors (CBT) are rare paragangliomas of the head and neck region arising from the carotid body. The location of the tumor in the neck region amidst vital neurovascular structures makes it challenging even for a vascular surgeon to excise it successfully without any complications. While intraoperative bleeding, post-operative/intraoperative stroke, and cranial nerve palsies remain the most common complications, carotid artery pseudoaneurysm following carotid body tumor excision (CBTE) is extremely rare. After an extensive search of the literature in PubMed, Embase, and Google Scholar using the keywords ‘carotid pseudoaneurysm’, and ‘Carotid body tumor excision complications’, it was determined that carotid artery pseudoaneurysm following CBTE has only been reported thrice in the literature.^1-3^ Carotid artery pseudoaneurysms are quite rare and usually occur secondary to trauma or iatrogenic injuries during head and neck surgeries. Having a potentially lethal outcome if untreated with complications ranging from bleeding, rupture into the trachea or pharynx, and cerebral embolism with resulting paresis, their prompt diagnosis is essential. They usually present with pulsatile neck mass with resulting mass effect onto the trachea, esophagus, and nerves causing compressive symptoms and nerve palsy. Endovascular management is presently the cornerstone of treatment, having the advantage of being less invasive, and is especially helpful whenever surgery may be contraindicated. However, there is a certain gap in knowledge regarding guidelines for the management of carotid artery pseudoaneurysms. Here we describe the occurrence and successful surgical management of a ruptured external carotid artery pseudoaneurysm following CBTE.

This manuscript was prepared in accordance with the Helsinki Declaration and CARE guidelines. Informed consent was taken from the patient before submission of this case report along with the images to the journal. Since it is a case report, no ethical clearance has been obtained.

## CASE REPORT

**Figure 1:**
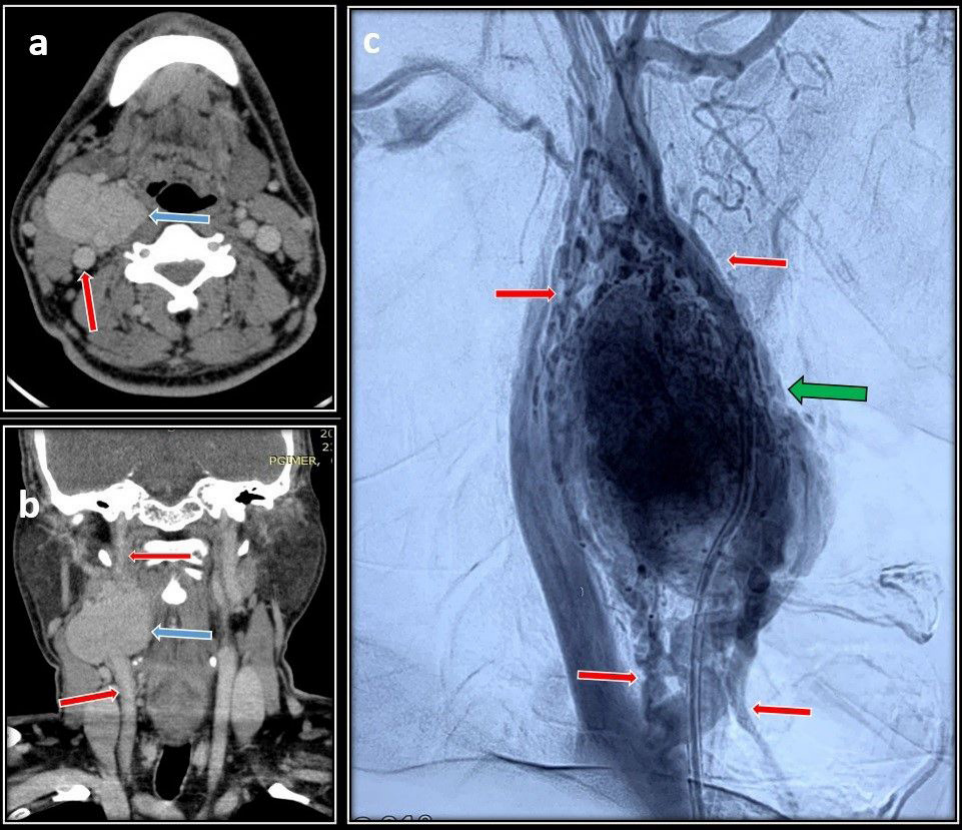
Pre-operative CT Angiography images showing a carotid body tumor (CBT) arising from the carotid artery bifurcation. **A)** Axial cut showing CCA (red arrow) and CBT (blue arrow); **B)** Coronal cut showing carotid artery and tumor. Preoperative angiography image showing multiple feeder vessels (red arrows) supplying the tumor (blue arrow); **C)** Digital Subtraction Angiography image showing the tumor(green arrow) supplied by feeder vessels( red arrow).

**Figure 2:**
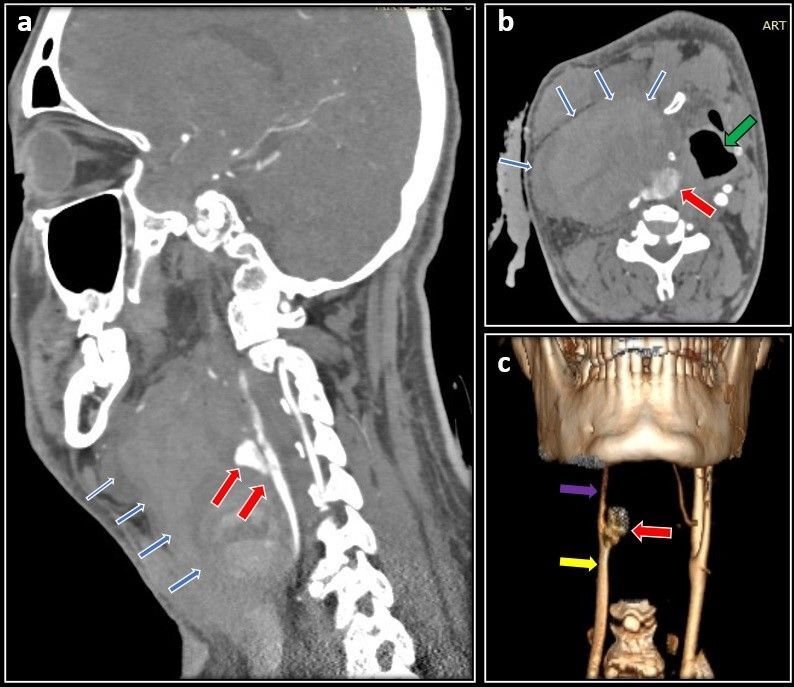
CT Angiography images after pseudoaneurysm formation. **A)** Sagittal cut showing a large hematoma (blue arrows) along with the pseudoaneurysm (red arrows) arising from the CCA; **B)** Axial cut showing the tumor blush (red arrow), and hematoma (blue arrows) causing severe compression of the trachea (green arrow); **C)** Reconstructed image shows the CCA (yellow arrow), attenuated ICA (violet arrow), and aneurysm (red arrow) at the carotid bifurcation with non-visualization of the ECA.

**Figure 3:**
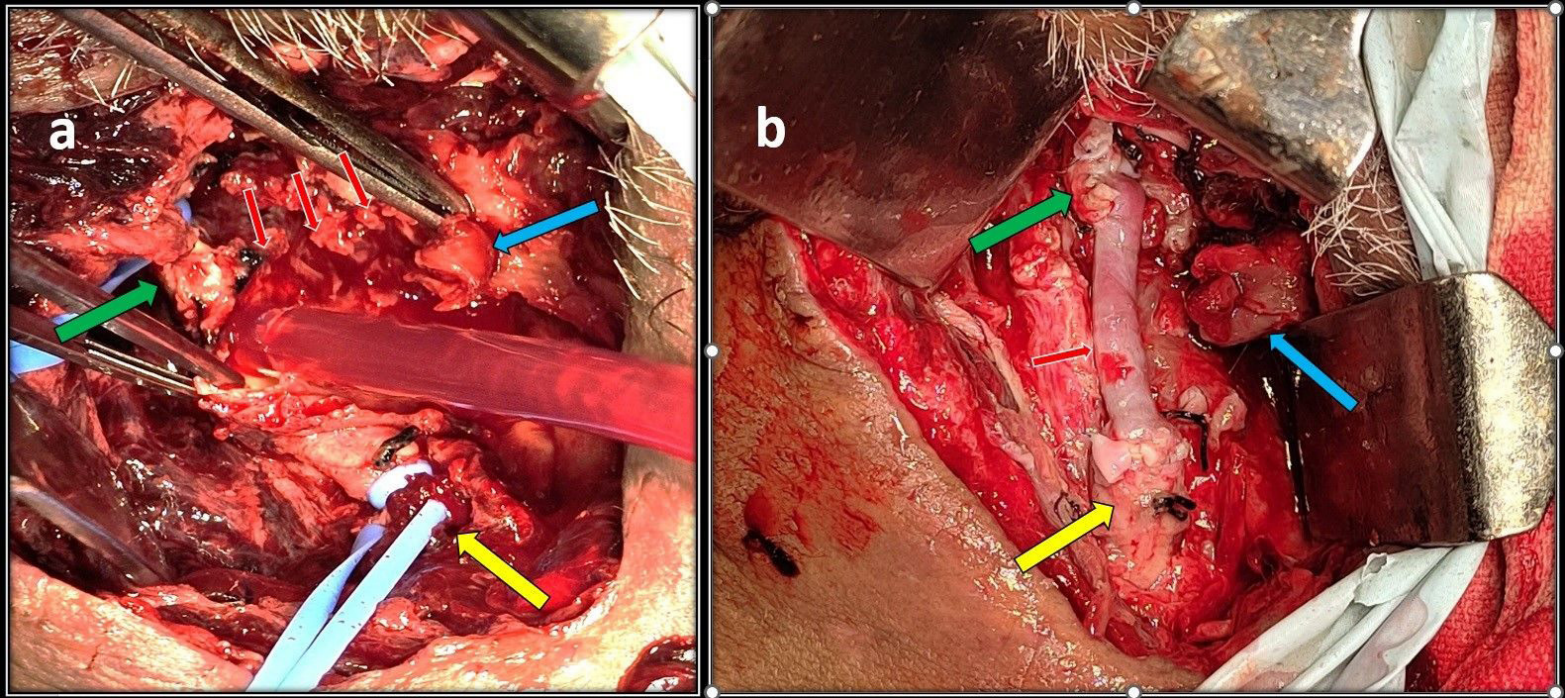
**A)** Intraoperative image showing CCA (yellow arrow), ICA (green arrow), and ECA (blue arrow) along with the pseudoaneurysm wall (red arrows); **B)** The vascular reconstruction image shows a saphenous vein graft (red arrow) interposed between the CCA (yellow arrow) and ICA (green arrow) along with ligated ECA STUMP (blue arrow).

**Figure 4:**
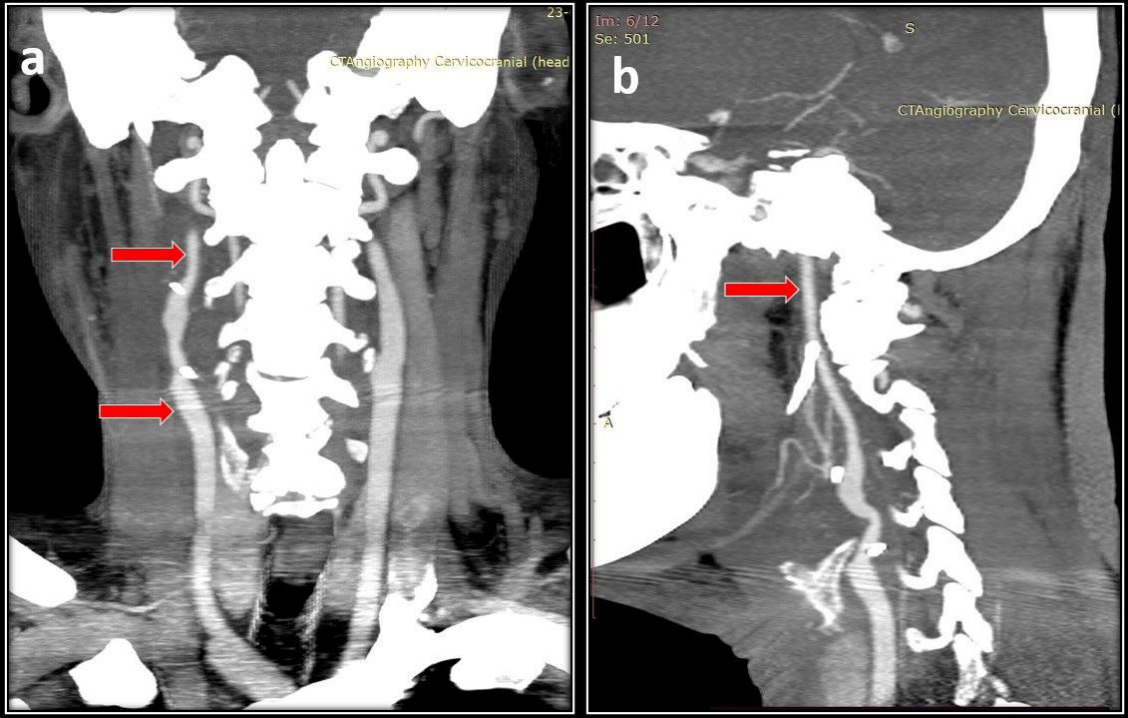
**A)** Postoperative follow-up CT angiography images showing a patent graft and **B)** patent circulation of the ICA.

A 47-year-old male presented with right-sided neck swelling for the past 10 years. Noticed incidentally by him, it gradually increased in size to approximately 5*5 centimeter (cm). The swelling was not associated with pain, headache, dysphagia, or any neurological deficits. Clinical examination revealed right-sided, pulsatile, firm swelling below the angle of the mandible of 5*5 cm in craniocaudal extent. Carotid pulsation was not palpable separately from the lesion. Computed Tomography (CT) Angiography of the neck showed a well-defined lesion measuring 3*5.5*4.7 cm extending from the bifurcation of the common carotid artery (CCA) up to the angle of the mandible splaying and encasing both the ICA and ECA up to 180^o^ (Shamblin type 2) (Figure 1A, 1B). Preoperative embolization done prior to surgery showed multiple tortuous feeder vessels from the ascending right pharyngeal artery (Figure 1C). Embolization of the feeder vessels was done 1 day before surgery and was uneventful. Intraoperatively, the tumor measuring 4*5*4 cm in size, was adherent to the CCA bifurcation, ECA, and ICA, and a sub adventitial excision was done. Multiple feeder vessels arising from the ECA were ligated with sutures and cauterized with bipolar. Post excision, the CCA, ICA, and ECA were healthy and pulsatile. Total blood loss was 200 ml and the duration of surgery was 2 hours. The patient did well post-surgery. There were no neurological deficits or cranial nerve palsy, he was discharged on postoperative day five, and histopathology confirmed a carotid body tumor. Ten days post-surgery, he presented with rapidly increasing swelling over the right side of the neck with dyspnea and dysphagia. He was tachycardic and tachypneic. A pulsatile swelling of size 10*8cm was present on the right side of the neck with deviation of the trachea to the left. Urgent CT angiography revealed a pseudoaneurysm measuring 2.6*1.6 cm arising from the distal CCA up to the bifurcation with active bleeding and hematoma measuring 7.7*5.5*10 cm causing mass effect over the proximal cervical ICA with severe luminal attenuation (Figure 2). The proximal ECA stump was not visualized. During the angiography itself, there was active bleeding from the neck wound for which compression was given and the patient was moved to the operating theater. He was intubated with the aid of a video laryngoscope and adequate ventilation was achieved. Intraoperatively, 200 ml of clots were present with active bleeding from the CCA bifurcation. Distal 1cm of the common carotid, the proximal ends of the ICA and ECA near the bifurcation were forming a pseudoaneurysm, which was sloughed off (Figure 3A). Bulldog clamps were initially applied to the sloughed ends of the CCA, ICA, and ECA to achieve hemostasis. Dissection was further continued along the CCA, ICA, and ECA till healthy walls were found. The ECA was ligated and a CCA to ICA bypass was done with a reversed saphenous vein graft (Figure 3B). The patient required ventilatory support immediately postoperatively and was extubated the next day. Postoperatively, marginal mandibular nerve palsy was evident, but no other neurological deficits were present. The patient did well in the postoperative period and was discharged on day 7. At 3 months follow up he is doing well and a follow-up CT showed a patent venous graft with well-maintained cerebral circulation (Figure 4).

## DISCUSSION

Carotid artery pseudoaneurysms are quite rare and usually result from traumatic blunt/penetrating injuries, retropharyngeal/parapharyngeal abscess, malignant invasion, post radiotherapy and iatrogenic injuries following radical neck dissection, needle puncture, or tonsillectomy.^4^ Unlike a true aneurysm, a pseudoaneurysm is devoid of all three vessel layers and thus is more often prone to rupture.^5,6^ Presenting as a pulsatile neck mass with dysphagia or dyspnea and neurological deficits resulting from compression on surrounding aero-digestive tract and cranial nerves, they have potentially fatal outcomes like rupture into an aerodigestive tract or intracranial embolism or occlusion and thus require urgent and immediate treatment.^7^

Though the incidence of CCA pseudoaneurysm is lower, the pathophysiology of its formation is known. Arterial injury causing disruption of intima and media resulting in dissection or thrombosis of CCA/ICA post-CBT excision is described and this can potentially lead to pseudoaneurysm formation.^4^ In our case, proximal CCA control and distal ECA and ICA control were taken with vessel loops prior to excision of the CBT and this handling might have led to the probable intimal-medial disruption. Although systemic anticoagulation is the standard treatment for dissection and spontaneous healing is known to occur in a significant proportion, a subset of patients can develop residual stenosis or pseudoaneurysm.^8^ The use of anticoagulation in patients with pseudoaneurysm may also be contraindicated because of the risk of bleeding. However, dissection occurring after the initial stage of arterial injury and anticoagulation at that particular time may be more beneficial with a lesser risk of bleeding at that time. Our patient, who presented at 10 days with ruptured CCA-ECA pseudoaneurysm, needed urgent exploration due to compressive symptoms and bleeding from the neck wound.

Pseudoaneurysm is diagnosed with color doppler showing the yin yang phenomenon and CECT angiography showing mass with intense enhancement projecting outside the confines of the arterial wall with or without mural thrombus or contrast leak depicting free rupture.^4^

The various treatment options are USG-guided compression, percutaneous thrombin injection, coil embolization, endovascular stent graft, and surgery.^3,9^ Although USG-guided compression has not been reported in CCA-ECA pseudoaneurysms due to a higher risk of rupture and thromboembolic events, reported treatment failures are associated with larger, multiloculated, wide-necked, and high flow rate pseudoaneurysms and with patients receiving high dose anticoagulation.^4^ Endovascular management with stent graft is presently the frontline treatment option for CCA pseudoaneurysm. Reduced invasivity, reduced hospital stays, and no need for general anesthesia have made it the foremost treatment in such cases. However, the decision regarding vessel preservation and parent artery occlusion (PAO) depends upon several factors such as etiology, site, age of patient, and status of collateral circulation.^10^ Use of stents, either bare or covered, remains the treatment of choice as it ensures patency of the parent artery with occlusion of the pseudoaneurysm. Reported complications are embolic stroke, dissection, thrombosis, rupture, and stent kinking.^11^

However, surgery may be the only option in cases of ruptured pseudoaneurysms with active bleeding, mass effect on the airway causing hemodynamic instability, and failed endovascular treatment. In our case, there was a ruptured pseudoaneurysm with active bleeding, a large hematoma causing compression and deviation of the trachea, and altered hemodynamics requiring urgent exploration, evacuation of hematoma, and bypass grafting. Although surgery is an effective treatment method, it is technically complicated and requires much experience and complications such as cranial nerve palsy, stroke, rupture during surgery, or leakage into the surgical area can occur. The rate of stroke or death during surgery is reported to be between 9 and 15%, and the incidence of cranial nerve injuries is reported to be as high as 15%.^10^ There are only 3 reported cases of carotid artery pseudoaneurysm post excision of carotid body tumor (Table 1) which were managed conservatively on anticoagulation,^3^ with stent-graft placement for a CCA pseudoaneurysm,^2^ and by re-exploration with the repair of ECA rent followed by parent artery occlusion for ruptured pseudoaneurysm at the CCA-ICA junction.^1^

**Table 1:** Details of Reported Carotid Artery Pseudoaneurysms post CBTE.

**Age/sex**	**Reference number**	**Shamblin type**	**Intra-op/post-op events**	**Post-op day of pseudoaneurysm presentation and details**	**Management**	**Complications/ follow up**
35/male	3	2	ICA partial thrombosis and dissection with stroke on POD1	POD- 14/ size 2.3* 1.5 cm pseudoaneurysm from ICA with 2mm neck	Conservative, small neck size and high risk for surgery	Recovered from the neurological deficit
65/male	2	3	None	POD-30/ICA Pseudoaneurysm	Stent grafting	None/ Recovered well
44/male	1	2	None	● POD-8/ Ligated ECA stump pseudoaneurysm	● Excision with ligation of ECA rent	Post-procedure stroke with prolonged hypotension. Discharged on day 55.
					●Endovascular with parent artery occlusion (PAO)	
				●Ruptured pseudoaneurysm at CCA-ICA junction at day 25		
47/male	Our case	2	None	POD 10/ distal CCA-ECA pseudoaneurysm 26* 16 mm with 7.7* 5.5* 10 cm hematoma	Surgery with ligation of ECA and CCA to ICA bypass vein graft	Recovered well without any neurological deficits

CBTE: Carotid body tumor excision; CCA: Common carotid artery; ICA: Internal carotid artery; ECA: External carotid artery.

To conclude, pseudoaneurysm formation following CBTE is extremely rare. The ideal management of pseudoaneurysm following CBTE is yet to be established. We opted for surgical management in our patient since he was hemodynamically unstable and had a large hematoma near the carotid which was causing compression of the trachea.
